# Effect of Substituents on the Homopolymerization Activity of Methyl Alkyl Diallyl Ammonium Chloride

**DOI:** 10.3390/molecules27154677

**Published:** 2022-07-22

**Authors:** Xu Jia, Xiujuan Zhang, Wenhui Peng, Kui Yang, Xiao Xu, Yuejun Zhang, Guixiang Wang, Xianping Tao

**Affiliations:** 1 School of Chemistry and Chemical Engineering, Nanjing University of Science and Technology, Nanjing 210094, China; zhxiujuan@njust.edu.cn (X.Z.); pwhawe@njust.edu.cn (W.P.); yangkui1123@163.com (K.Y.); xuxiao@njust.edu.cn (X.X.); zhyuejun@njust.edu.cn (Y.Z.); wanggx1028@163.com (G.W.); 2 National Quality Supervision Testing Center for Industrial Explosive Materials, Nanjing University of Science and Technology, Nanjing 210094, China; 3 Nantong Professional Institute, College of Chemical and Biological Engineering, Nantong 226007, China; taoxianping@163.com

**Keywords:** methyl alkyl diallyl ammonium chloride, quantum chemical calculation, homopolymerization, polymerization activity, polymerization rate, kinetic

## Abstract

Among nitrogen-containing cationic electrolytes, diallyl quaternary ammonium salt is a typical monomer with the highest positive charge density, which has attracted the most attention, especially in the research on homopolymers and copolymers of dimethyl diallyl ammonium chloride (DMDAAC), which occupy a very unique and important position. In order to improve the lipophilicity of substituted diallyl ammonium chloride monomers under the premise of high cationic charge density, the simplest, most direct, and most efficient structure design strategy was selected in this paper. Only one of the substituents on DMDAAC quaternary ammonium nitrogen was modified by alkyl; the substituents were propyl and amyl groups, and their corresponding monomers were methyl propyl diallyl ammonium chloride (MPDAAC) and methyl amyl diallyl ammonium chloride (MADAAC), respectively. The effect of substituent structure on the homopolymerization activity of methyl alkyl diallyl ammonium chloride was illustrated by quantum chemical calculation and homopolymerization rate determination experiments via ammonium persulfate (APS) as the initiator system. The results of quantum chemistry simulation showed that, with the finite increase in substituted alkyl chain length, the numerical values of the bond length and the charge distribution of methyl alkyl diallyl ammonium chloride monomer changed little, with the activation energy of the reactions in the following order: DMDAAC < MPDAAC < MADAAC. The polymerization activities measured by the dilatometer method were in the order DMDAAC > MPDAAC > MADAAC. The activation energies *E*_a_ of homopolymerization were 96.70 kJ/mol, 97.25 kJ/mol, and 100.23 kJ/mol, and the rate equation of homopolymerization of each monomer was obtained. After analyzing and comparing these results, it could be easily found that the electronic effect of substituent was not obvious, whereas the effect of the steric hindrance was dominant. The above studies have laid a good foundation for an understanding of the polymerization activity of methyl alkyl diallyl ammonium chloride monomers and the possibility of preparation and application of these polymers with high molecular weight.

## 1. Introduction

Water-soluble cationic polyelectrolytes are a kind of important polymers, among which the study of homopolymers and copolymers of dimethyl diallyl ammonium chloride (DMDAAC) occupies a very unique and important position. They have been widely used in water treatment, petroleum exploitation, textile printing, dyeing, the daily chemical industry, the pharmaceutical chemical industry, electrochemical materials, electronic sensors, and other fields [1,2,3,4,5,6,7,8,9]. In addition, to improve the relative molecular weight of DMDAAC-based polymers to form products with serial cationicity and relative molecular weight, in recent years, it was found that the group structure of quaternary ammonium nitrogen is directly related to its mode of action and efficiency at the phase interface, especially in some lipophilic application fields. The application performance of DMDAAC polymers is not satisfactory [10,11,12], with large room for improvement.

Various factors can affect the monomer polymerization activity, properties, and performance of polymer. Researchers have reached a consensus that the monomer substituents are the most fundamental factor [13,14,15,16,17,18,19,20]. For example, Malldavi et al. [21] synthesized N-substituted methyl alkyl diallyl ammonium iodide monomer and its polymer. Timofeeva et al. [10] synthesized N-substituted diallyl ammonium trifluoroacetate monomer and its polymer. Olsson et al. [16] synthesized N-substituted diallyl azacycloalkane quaternary ammonium salt monomer and its polymer. Wang et al. [22] synthesized novel non-crosslinked and crosslinked, hydrophobically modified homo- and copolymers using alkyl methyl diallyl ammonium chloride monomers. Although the above studies have greatly expanded the species of diallyl quaternary ammonium monomers and polymers, they are not conducive to the acquisition of polymerization products with high molecular weight, due to the excessive influence on the electron cloud density of allyl bonds or the large steric hindrance caused by the modified functional groups, resulting in an unsatisfactory polymerization reaction rate and a low relative molecular weight of the polymers [23,24,25]. Therefore, the structural modifications of these monomers and the corresponding polymers are limited [21,26].

**Figure 1:**
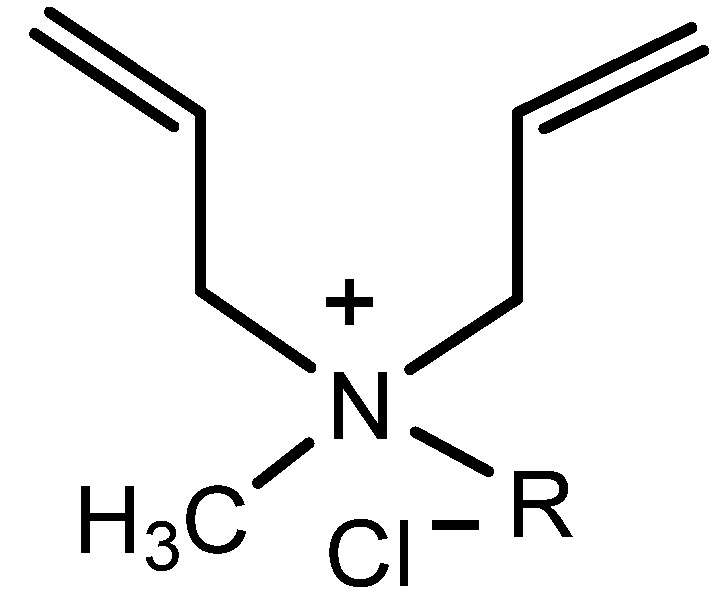
General structure of methyl alkyl diallyl ammonium chloride.

Methyl alkyl diallyl ammonium chloride is such a modified monomer which was obtained by replacing a methyl group on DMDAAC quaternary ammonium nitrogen with an alkyl group with a finite-length carbon chain [27,28]. Although the steric hindrance increased, it theoretically had no negative effect on the electron cloud density of the allyl double bond. Obviously, this is the best strategy to improve the lipophilicity of monomers with minor effects on the polymerization activities of the monomers under the premise of maintaining their high positive charge density. However, little attention has been paid to the effect of the alkyl substituents on the activity of polymerization. The general structure of methyl alkyl diallyl ammonium chloride is shown in Figure 1. 

This paper takes methyl alkyl diallyl ammonium chloride as the research object, with propyl and amyl as the substituents. Firstly, density functional theory (DFT) was used to optimize the stable figuration parameters (including bond length, bond angle, and charge distribution) of a series of monomers at the B3LYP/6-31G* level, and the initial structures of monomers for DFT were taken as the structures with the most stable and lowest energy as optimized at the B3LYP/6-31G* level. Then, the activation energy of primary free radicals formed by quaternary ammonium monomers was calculated. The parameter values were calculated for the formation of monomer primary free radicals initiated by initiator free radicals in the process of free-radical polymerizations. Since the DFT calculation in vacuum does not consider the influence of solvent, the simulation results could only approximate the theoretical influence law excluding the solvent effect. Furthermore, the kinetics of homopolymerization of each monomer via an ammonium persulfate (APS) initiator were studied using the dilatometer method. The apparent activation energy *E*_a_ and the rate equation of polymerization of each monomer were determined. Therefore, the polymerization activities of each monomer and dimethyl diallyl ammonium chloride were compared according to the kinetic parameters of homopolymerization. This work systematically studied and revealed the structure–activity relationship of the monomers by means of theoretical calculation and experiment. The results allow building a solid theoretical and experimental foundation for structure expansion, polymerization research, and application development of substituted allyl quaternary ammonium salt monomers and their polymers.

## 2. Results

### 2.1. Monomer Conformation Optimization

**Figure 2:**
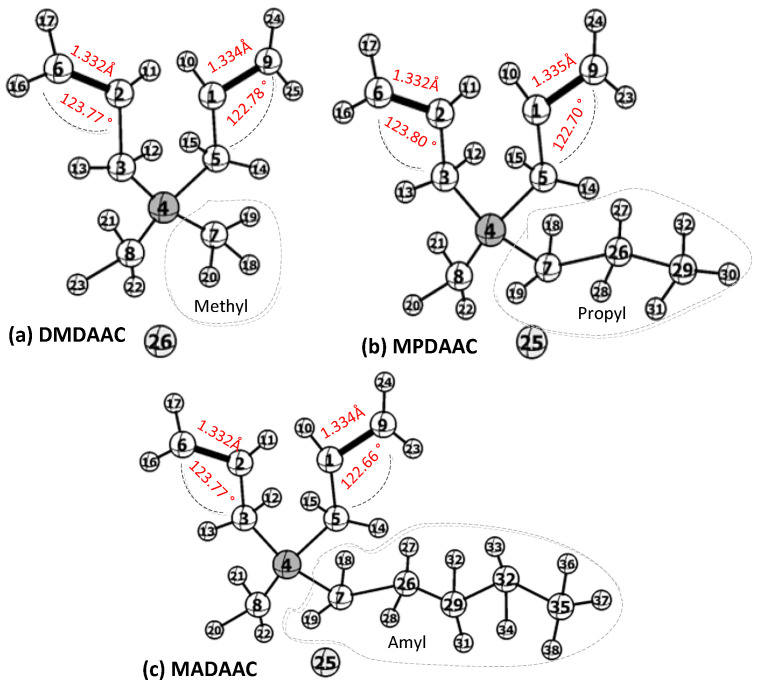
The optimized structures of methyl alkyl diallyl ammonium chloride monomers at the B3LYP/6-31G* level. Stable conformation of DMDAAC (**a**), stable conformation of MPDAAC (**b**), stable conformation of MADAAC (**c**), nitrogen atom (4), chlorine atom (25,26), carbon atom (1,2,3,5,6,7,8,9), and hydrogen atoms (remainder).

The stable conformations of DMDAAC (Figure 2a), MPDAAC (Figure 2b), and MADAAC (Figure 2c) were optimized at the B3LYP/6-31G* level. The geometric parameters of the optimized structures are shown in Table 1. The xyz-coordinates of all the optimized structures are shown in supplementary materials (Tables S1–S3).

The structural characterization results on the monomers are shown in the supplementary materials (Figures S1–S9).

According to the comparison of the optimized conformation parameters of DMDAAC (Figure 2a), MADAAC (Figure 2b), and MPDAAC (Figure 2c), as well as the results of quantum chemical calculation (Table S4), on the basis of the DMDAAC monomer structure, although the alkyl chain lengths of methyl alkyl diallyl ammonium chloride monomers increased, the bond lengths and bond angles between C–C double bonds were basically unchanged, and the bond lengths of allyl double bonds –C2=C6– and –C1=C9– of the monomers were basically maintained at about 1.332 Å and 1.334 Å. The bond angles of allyl double bonds –C3–C2=C6– and –C5–C1=C9– were maintained in the range of 123.77° to 123.80°and 122.66° to 122.78°, respectively. With the increase in the number of C atoms in the substituents, the bond lengths and bond angles of alkyl substituent were basically unchanged, at about 1.54 Å and 109.58°, respectively.

On the other hand, Mulliken charges were used for atomic charge analysis, and the charge distributions of a series of methyl alkyl diallyl ammonium chloride monomers were obtained. The results of charge distribution of methyl alkyl diallyl ammonium chloride show that whether DMDAAC, MPDAAC, or MADAAC was the monomer (Table S5), H atoms were positively charged, while negative charges were concentrated on Cl, N, and C atoms. In the series of monomer molecules, with the increase in the length of alkyl substituent chains on quaternary ammonium nitrogen, the electron cloud density of the double bonds of the monomer molecule changed slightly, which would lead to a change in the bond polarity. At the same time, the alkyl substituents were all electron donor groups, and their electron-supplying capacity was in the order of methyl < propyl < amyl. Due to the difference in the electron-supplying/absorbing ability of alkyl substituents, the allyl double bond electron cloud moved in a certain direction. This might affect the activity of radical polymerization of the allyl double bond and the stability of the products [29]. However, there was little difference in electronegativity among alkyl substituents; hence, the effect of alkyl chain length on the electronic effect of monomers was not obvious, which could also be confirmed from the comparison of the electron density data of the allyl C–C double bonds –C2=C6– with –C1=C9–, whose charge distribution did not change much (approximately −0.068, −0.314, −0.051, and −0.338, respectively). The electronegativities of Cl and N atoms remained basically unchanged with the increase in the length of substituted alkyl groups, which were about −0.74 and −0.37, respectively.

From the calculations in Table 1, it can be known that the order of activation energy of the reaction between methyl alkyl diallyl ammonium chloride monomers and the APS initiator was DMDAAC < MPDAAC < MADAAC, while the order of polymerization activity of each monomer was DMDAAC > MPDAAC > MADAAC. The largest difference in activation energy was between DMDAAC and MADAAC, i.e., 17.06 kJ/mol.

In this paper, the calculation of the activation energy of the reaction between the monomer polymerization and initiator radical to form the monomer primary radical took into account the comprehensive influence of the electronic effect and space effect, and the difference in activation energy obtained on this basis was not obvious. In order to measure and compare the effect mechanism of alkyl substituents on the polymerization of methyl alkyl diallyl ammonium chloride, it was necessary to perform a direct investigation using the homopolymerization kinetics and to conduct a comprehensive evaluation combined with theoretical calculations.

### 2.2. Determination of Activation Energy of Polymerization of Monomers

**Figure 3:**
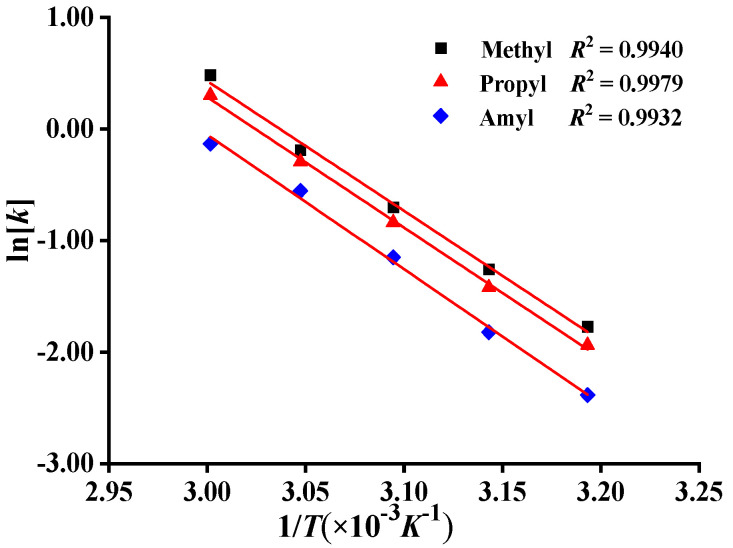
Relationship between ln*k* and 1/*T*.

Under the condition of low conversion (<10%), the slope of the fitting curve of the conversion of each monomer with respect to the reaction time increased with the increase in polymerization temperature (Figure S10). This was consistent with the effect of polymerization temperature on the polymerization rate, i.e., with the increase in polymerization temperature, the polymerization rate increased, and the conversion increased. Then, using 1/T as abscissa and lnk as ordinate, linear regression analysis was carried out to get the dependence curve as shown in Figure 3, and the coefficient of determination was *R^2^* > 0.99, showing a good linear relationship between lnk and 1/T. The polymerization rate of each monomer increased with the increase in temperature in the range of 40 °C to 60 °C. The activation energies of polymerization of different monomers were obtained (Table 2). The slopes of the fitting curve of lnk versus 1/T of DMDAAC, MPDAAC, and MADAAC were −11.629, −11.696, and −12.054, respectively, and the polymerization activation energies *E*_a_ of DMDAAC, MPDAAC, and MADAAC were 96.70 kJ/mol, 97.25 kJ/mol, and 100.23 kJ/mol, respectively, which were obtained using the Arrhenius equation. At the same time, the polymerization activation energy of DMDAAC was 96.70 kJ/mol, higher than the 71.99 kJ/mol recorded in the literature [30], which might be due to the differences in monomer concentration, initiator concentration, and reaction temperature.

### 2.3. Determination of Monomer Concentration Iindices

**Figure 4:**
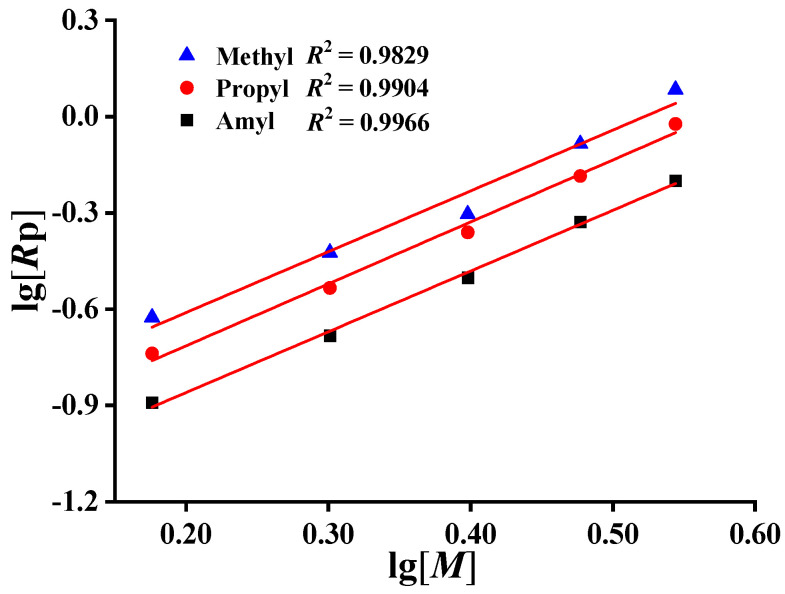
Relationship between lg *R*_p_ and lg [*M*].

Under the condition of low conversion (<10%), the conversion was approximately proportional with time (Figure S11), indicating that the reactions were approximately uniform. With the increase in DMDAAC monomer concentration, the slopes of the fitting curve of conversion versus reaction time increased, which was consistent with the effect of monomer concentration on the polymerization rate, indicating that with the increase in monomer concentration, the polymerization rate and the conversion increased. Using lg [*M*] as the abscissa and lg *R*_p_ as the ordinate, linear regression analysis was carried out (Figure 4), revealing a coefficient of determination *R^2^* > 0.98, which showed the good linear relationship between lg *R*_p_ and lg [*M*]. The polymerization rate of each monomer increased with the increase in monomer concentration in the range of 1.5 mol/L to 3.5 mol/L, and the slopes of lg *R*_p_ versus lg [*M*] of different monomers were obtained (Table 3). When the reaction temperatures and initiator concentrations were constant, the slopes of lg *R*_p_ versus lg [*M*] of DMDAAC, MPDAAC, and MADAAC were 1.92, 1.93, and 1.89, respectively, which showed that the relationships between polymerization rate and monomer concentration were *R*_p1_ ∝ [*M*]^1.92^, *R*_p2_ ∝ [*M*]^1.93^, and *R*_p3_ ∝ [*M*]^1.89^, respectively. The monomer concentration indices of methyl alkyl diallyl ammonium chloride were much higher than the theoretical value of 1, which might be due to the fact that the process of generating monomer free radicals via the reaction between the initial free radical and the monomer was not very fast, and the initiation rate could be determined not only by the decomposition rate of the initiator, but also by the concentration of the monomer in the second step. This might be due to the complex interaction between monomers [19].

### 2.4. Determination of Concentration Indices of Initiator

**Figure 5:**
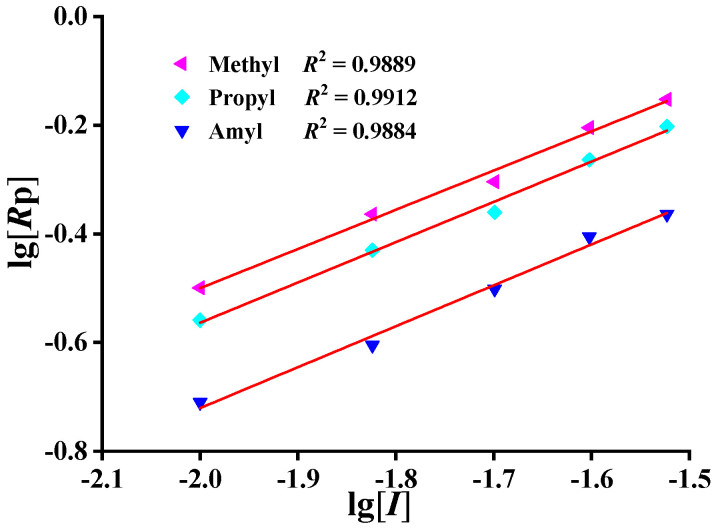
Relationship between lg *R*_p_ and lg [*I*].

Under the condition of low conversion (<10%), the conversion was approximately proportional to time (Figure S12), indicating that the reactions were approximately uniform. The slopes of the fitting curves of conversion versus reaction time increased with the increase in initiator concentration, which was consistent with the relationship between the initiator concentration and the polymerization rate, indicating that the polymerization rate and the conversion increased with the increase in initiator concentration. Using lg [*I*] as the abscissa and lg *R*_p_ as the ordinate, linear regression analysis was carried out to get the relation curve (Figure 5). The coefficient of determination *R^2^* > 0.98 showed that there was a good linear relationship between lg *R*_p_ and lg [*I*]. The polymerization rate of each monomer increased with the increase in initiator concentration in the range of 0.01 mol/L to 0.03 mol/L. The slopes of lg *R*_p_ versus lg [*I*] of different monomers are shown in Table 4. When the reaction temperature and monomer concentration were constant, the slopes of lg *R*_p_ versus lg [*I*] of DMDAAC, MPDAAC, and MADAAC were 0.72, 0.74, and 0.75, respectively, which showed that the relationships between polymerization rate and initiator concentration were *R*_p1_ ∝ [*I*]^0.72^, *R*_p2_ ∝ [*I*]^0.74^, and *R*_p3_ ∝ [*I*]^0.75^. The reaction order of each monomer initiator concentration was higher than the theoretical value of 0.5, indicating that the chain termination mode featured the coexistence of double-base termination and single-base termination.

## 3. Discussion

### 3.1. The Effect of Substituents on the Conformation Parameters of Monomer Molecules

Through the quantum chemical structure optimizations of methyl alkyl diallyl ammonium chloride and the calculations of homopolymerization activation energies of the monomers after optimized conformation, the molecular conformation parameters of allyl double bonds and the activation energies of primary radical homopolymerization of DMDAAC, MPDAAC, and MADAAC were obtained (Table 5). It can be seen from Table 5 that the effects of different alkyl substituents on the molecular conformations of methyl alkyl diallyl ammonium chloride were not apparent, and there were minor changes with the bond length and bond angle upon the increase of C atoms in the carbon chain. In particular, the activation energy required for the formation of primary free radicals by methyl alkyl diallyl ammonium chloride monomers gradually increased with the increase in the length of the alkyl chain, which means that the polymerization difficulty of monomers increased gradually. The monomer order of polymerization activities of methyl alkyl diallyl ammonium chloride was DMDAAC > MPDAAC > MADAAC. A longer alkyl substituent chain length denoted greater difficulty to form the monomer primary radical and a lower monomer polymerization activity.

The results were basically consistent with the design scheme, indicating that the introduction of alkyl substituents mainly affected the spatial structure of methyl alkyl diallyl ammonium chloride molecules, but the impacts of the electron cloud density of each atom in the molecule were limited. Although each alkyl substituent was an electron donor group, there was little difference in electronegativity.

### 3.2. Effect of Substituents on the Activity of Monomer Polymerization

Combined with the experimental results of the kinetics of polymerization, when the basic polymerization conditions were as follows: [*M*] = 2.5 mol/L, [*I*] = 0.02 mol/L, and polymerization temperature T = 50 °C, and the substituents were methyl, propyl, and amyl, the polymerization rate equation and the apparent activation energy of polymerization of methyl alkyl diallyl ammonium chloride were calculated (Table 6). The effects of substituents on the polymerization activity of monomers could be determined from the polymerization rate *R*_p_, polymerization kinetic parameter *k*_p_/*k*_t_^1/2^, and polymerization activation energy *E*_a_.

#### 3.2.1. Polymerization Reaction Rate *R*_p_ Comparison of Monomer Polymerization Activity

As shown in Table 7, the order of polymerization rates under the same polymerization conditions from high to low was *R*_p1_ > *R*_p2_ > *R*_p3_. According to the comparison of polymerization rates of methyl alkyl diallyl ammonium chloride, the order of polymerization activities was DMDAAC > MPDAAC > MADAAC, indicating that the polymerization activities of methyl alkyl diallyl ammonium chloride decreased with the increase in alkyl chain length [31,32]. In addition, since the reaction solvent was water, the hydrophobicity of the substituent would also affect the polymerization rate *R*_p_. A longer alkyl chain of the substituent resulted in stronger hydrophobicity and a lower *R*_p_.

#### 3.2.2. Calculation of *k*_p_/*k*_t_^1/2^ and Comparison of Polymerization Activities of Different Monomers

The polymerization kinetics of methyl alkyl diallyl ammonium chloride were studied. Since the initiator concentrations and polymerization temperatures were the same, the decomposition rate constant of the initiator was the same. The value of *k* can be compared by calculating *k*_p_/*k*_t_^1/2^. The value of *k* was calculated using Equation (1).

(1) $${\;R}_{p}=k\;{\left[I\right]}^{\frac{1}{2}}{\;[M]=k}_{p}\;(\frac{fk_{d}}{k_{t}}{)}^{n}\;{\left[I\right]}^{n}\;\left[M\right]{\;}^{m},$$

where *k*_p_, *k*_t_, and *k*_d_ are chain growth rate constant, initiator decomposition rate constant, and chain termination rate constant, respectively.

When the polymerization process conditions were [*M*] = 2.5 mol/L, [*I*] = 0.02 mol/L, and T = 50 °C, the calculated results were as shown in Table 8, with the *k* values of methyl alkyl diallyl ammonium chloride in the order *k*_1_ > *k*_2_ > *k*_3_. Accordingly, the order of polymerization activities was DMDAAC > MPDAAC > MADAAC, indicating that the polymerization activities of methyl alkyl diallyl ammonium chloride decreased with the increase in alkyl chain length [29].

#### 3.2.3. Comparison of Polymerization Activities of Different Monomers by Activation Energies *E*_a_

According to $k=Ae^{-\frac{Ea}{RT}}$ and *E*_a_ = *E*_ad_ + *E*_ap_ – 1/2*E*_at_, *E*_ad_, *E*_ap_, and *E*_at_ were the decomposition activation energy of the initiator, the activation energy of the chain growth reaction, and the activation energy of the chain termination reaction, respectively. The total activation energy *E*_a_ was positive, indicating that, with the increase in temperature, the rate constant *k* increased, and the total polymerization rate also increased. A higher total activation energy *E*_a_ resulted in a greater effect of temperature on the polymerization rate [33].

From Table 6, it can be concluded that *E*_a1_ < *E*_a2_ < *E*_a3_, indicating that a longer alkyl chain led to a greater apparent activation energy of polymerization. The results showed that, with the increase in alkyl chain length, the difficulty of the formation of monomer free radicals increased and the polymerization activity decreased, which was the same as the polymerization rate.

The calculations of quantum chemistry were consistent with the results of the rate of monomer polymerization, indicating that the polymerization activities of monomer decreased with the increase in alkyl chain length. The increase in alkyl chain mainly enhanced the spatial effect of the alkyl chain. With the increase in alkyl chain length, the polymerization activities of methyl alkyl diallyl ammonium chloride decreased, which was mainly due to the increase in the substituent space effect, and the difficulty of the reaction of polymer segment free radicals with monomers increased [34].

### 3.3. Effect of Micelle Formation on the Activity of Monomer Polymerization

According to previous work [35], the surface activity information of the monomer including the critical micelle concentration (CMC), the surface tension (γCMC), and the negative logarithm of the concentration required to reduce the surface tension of water by 20 mN/m (pC_20_) were obtained. It is known that, with the increase in alkyl chain length of substituents, the surface activity of monomers increased, which makes it easier for the monomer to form micelles. When monomer micelles are formed, it is not conducive to the progress of the polymerization reaction. The result was that, as the chain length of the alkyl substituent increased, the polymerization reaction rate Rp became smaller, and the polymerization activation energy *E*_a_ became larger.

## 4. Materials and Methods

### 4.1. Materials

Ammonium persulfate (APS) (mass fraction ≥ 99.0%) (Sinopharm Chemical Pharmaceutical Co., Ltd., Shanghai, China), tetrasodium ethylenediaminetetraacetic acid (Na_4_EDTA) (mass fraction ≥ 99.0%) (Sinopharm Chemical Reagent Co., Ltd., Shanghai, China), sodium chloride (NaCl) (mass fraction ≥ 99.5%) (Shanghai Sinopharm, Shanghai, China), and nitrogen (purity 99.9%) (Nanjing Wenda Special Gas Co., Ltd., Nanjing, China) were obtained analytically pure. A capillary dilatometer (0.5–0.6 mm Ubbelohde viscometer) and mercury calibration volume (Shanghai Shenyi Glass Products Co., Ltd., Shanghai, China) were also used.

### 4.2. Quantum Chemical Calculation Results

**Figure 6:**
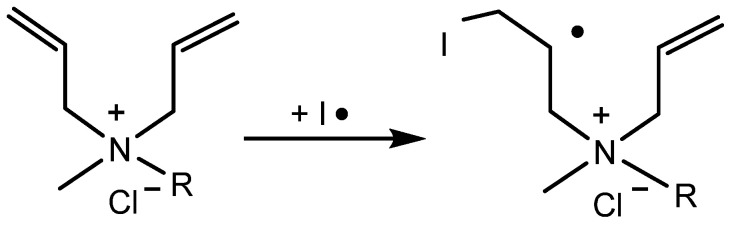
Primary radicals of monomers generated by radical reaction of methyl alkyl diallyl ammonium chloride monomers with APS initiator.

Dimethyl diallyl ammonium chloride (DMDAAC), methyl propyl diallyl ammonium chloride (MPDAAC), and methyl amyl diallyl ammonium chloride (MADAAC) were stably configured by quantum chemistry density functional theory (DFT) at the level of B3LYP/6-31G* [36,37,38,39], and the geometric parameters of the optimized structures were obtained. Then, the atomic charges were analyzed according to the Mulliken charge, and the charge distributions were obtained [40,41]. Furthermore, the molecular structures of the monomers were optimized and frequency-analyzed to obtain the minimum points on the potential energy surface. Then, the stable structure of the transition state of the monomers with the APS initiator radical (–SO_4_^−^) were found, and their activation energies were simulated. The above results could help to illustrate the relationships among the molecular reactants, transition states, and polymerization products of the monomers [42,43]. The reaction formula of the monomer primary radical formed by the reaction of methyl alkyl diallyl ammonium chloride monomers is shown in Figure 6. All the above simulations were carried out using the Gaussian 03 program.

### 4.3. Kinetics of Homopolymerization

#### 4.3.1. Experimental Methods

A certain amount of monomer was weighed and dissolved into different concentrations in a 50.0 mL capacity flask. Then, the capacity flask was put into a three-port flask of 100.0 mL volume with a stirrer, and the monomer solution was stirred at room temperature in nitrogen atmosphere for 20 min. After that, the required initiator was weighed and put into the three-port flask. The reaction solution was obtained after stirring for 10 to 15 min.

The reaction solution was poured into an ampere bottle in a dilatometer. When there were no air bubbles left after the capillary was plugged, the dilatometer was placed into a constant-temperature water bath, and the initial height of the liquid surface *h*_0_ was recorded. The reaction was stopped when the liquid level rose to a certain height *h*, and then the reaction mixture was quickly taken out and poured into 20 mL of cooled deionized water. The monomer conversion (i.e., *Conv.*%) was determined using the KBr/KBO_3_ method, and the *K* value was calculated according to Equation (2) [12].

(2) $$Conv.\%=\frac{\Delta V}{V_{0}}\frac{1}{K}=\frac{A\Delta h}{V_{0}}\;\frac{1}{K},$$

where *V*_0_ is the initial volume of the polymerization system, and *K* is the volume shrinkage factor.

#### 4.3.2. Determination of Polymerization Rate Equation

The polymerization rate *R*_p_ refers to the amount of polymer transformed by monomer per unit time, and the time *t* of ∆*h* was measured in the experiment. Under a low conversion rate (<10%), the *Conv.* vs. *t* diagram was obtained according to Equation (2). Changing the monomer [*M*] or initiator [*I*] concentration, the *R*_p_ under different conditions was derived by numerical differential interpolation. The slopes of the fitting curve of ln*R*_p_ versus ln[*M*] or ln[*I*] was the reaction order *m* or *n*. The specific process is shown in Equation (3) [44,45].

(3) $$\mathrm{In}R_{p}=\mathrm{In}k+m\mathrm{In}\left[M\right]+n\mathrm{In}\left[I\right],$$

where *R*_p_ is the total rate of polymerization, [*M*] is the concentration of monomer, [*I*] is the concentration of initiator, and *K* is the reaction rate constant.

#### 4.3.3. Determination of Apparent Activation Energy *E*_a_

The activation energy reflects the polymerization activities of monomers. The activation energy of each monomer was determined to compare the polymerization activity of each monomer. Equation (4) was obtained according to the *Arrhenius* equation [42].

(4) $$k=Ae^{-\frac{Ea}{RT}},$$

where *k* is the general rate constant, *A* represents the frequency factor, *R* is the gas constant, and *E*_a_ is the activation energy.

The monomer conversion (*Conv.*%) was determined using the KBr/KBO_3_ method. Then, the *K* value was calculated according to the expansion formula. The relation curve of *Conv.*% vs. *t* was drawn at different polymerization temperatures, and then the corresponding general rate constant *k* was calculated. The activation energy (*E*_a_) of polymerization could be calculated using the slope of the straight line which was calculated by linear regression from the diagram of ln*k* versus 1/*T*.

## 5. Conclusions

In this work, the monomer structures of DMDAAC, MPDAAC, and MADAAC were optimized by quantum chemistry, and the homopolymerization kinetics of methyl alkyl diallyl ammonium chloride were determined using the expansive agent method taking ammonium persulfate as the initiator. Moreover, the effects of different substituents on the polymerization activity of methyl alkyl diallyl ammonium chloride were studied.

The experimental results showed that, with the increase in carbon chain length of the substituents, the bond length, bond angle, and charge distribution of monomers did not change the results much. The monomeric order of the activation energies with APS initiator radical was DMDAAC < MPDAAC < MADAAC. The monomeric order of polymerization activities was DMDAAC > MPDAAC > MADAAC. The rate equations of polymerization were *R*_p1_ = *k*_1_ [*I*]^0.72^ [*M*]^1.92^, *R*_p2_ = *k*_2_ [*I*]^0.74^ [*M*]^1.93^, and *R*_p3_ = *k*_3_ [*I*]^0.75^ [*M*]^1.93^, respectively. Under the same conditions, the order of polymerization rates was *R*_p1_ > R_p2_ > *R*_p3_. The activation energies were *E*_a1_ = 96.70 kJ/mol^−1^, *E*_a2_ = 97.25 kJ/mol^−1^, and *E*_a3_ = 100.23 kJ/mol^−1^.

These results clarified that the theoretical calculation was consistent with the experimental results of monomer homopolymerization. The polymerization activities of methyl alkyl diallyl ammonium chloride decreased with the increase in the length of the alkyl chain, which was largely due to the enhancement of the spatial effect of substituents.

## Figures and Tables

**Table 1:** The total reactant energy *E*_R_, total energy *E*_(TS)_ in transition state, and activation energy (*E*_a_) in transition state for the reaction of methyl alkyl diallyl ammonium chloride monomers with APS initiator radical.

Monomer	Substituent	*E*_(R)_ (kJ·mol^−1^)	Zero-Point Energies (R)	*E*_(TS)_ (kJ·mol^−1^)	Zero-Point Energies (TS)	*E*_a_ (kJ·mol^−1^)
DMDAAC	Methy	−1528.4278	0.2463	−1528.4141	0.2483	42.53
MPDAAC	Propyl	−1607.0537	0.3039	−1607.0351	0.3051	51.98
MADAAC	Amyl	−1685.6829	0.3623	−1685.6673	0.3629	59.59

**Table 2:** Experimental results of measuring the polymerization activation energy *E*_a_ of different monomers.

Monomer	Substituent	Slope	*R* ^2^	*E*_a_ (kJ·mol^−^^1^)
DMDAAC	Methyl/R_1_	−11.629 ± 0.450	0.9940	96.70 ± 3.74
MPDAAC	Propyl/R_2_	−11.696 ± 0.268	0.9979	97.25 ± 2.23
MADAAC	Amyl/R_3_	−12.054 ± 0.413	0.9932	100.23 ± 3.43

**Table 3:** Experimental results of fitting curve slopes of lg *R*_p_ versus lg [*M*] of different monomers.

Monomer	*R*_p_	Slope	*R* ^2^
DMDAAC	*R*_p1_	1.89 ± 0.17	0.9829
MPDAAC	*R*_p2_	1.93 ± 0.09	0.9904
MADAAC	*R*_p3_	1.89 ± 0.06	0.9966

**Table 4:** Experimental results of fitting curve slopes of lg *R*_p_ versus lg [*I*] of different monomers.

Monomer	*R*_p_	Slope	*R* ^2^
DMDAAC	*R*_p1_	0.72 ± 0.04	0.9889
MPDAAC	*R*_p2_	0.74 ± 0.04	0.9912
MADAAC	*R*_p3_	0.75 ± 0.04	0.9884

**Table 5:** The molecular conformation parameters of allyl double bonds and the activation energies of primary radical homopolymerization of DMDAAC, MPDAAC, and MADAAC.

Monomer	*E*_a_ (kJ·mol^−1^)	C(9)-C(1)/C(6)-C(2)		C(5)-C(1)-C(9)/C(3)-C(2)-C(6)
		Length (Å)	Charge Density	Angle (°)
DMDAAC	42.53	1.33/1.33	−0.338, −0.051/−0.313, −0.068	123.77/122.78
MPDAAC	51.98	1.33/1.33	−0.339, −0.051/−0.315, −0.068	123.80/122.71
MADAAC	59.59	1.33/1.33	−0.339, −0.051/−0.314, −0.068	123.77/122.66

**Table 6:** Polymerization rate equation and the activation energy of polymerization of monomer.

Monomer	Substituent	Polymerization Rate Equation	*E*_a_ (kJ·mol^−1^)
DMDAAC	Methyl	*R*_p1_ = *k*_1_ [*I*]^0.72^ [*M*]^1.92^	96.70 ± 3.74
MPDAAC	Propyl	*R*_p2_ = *k*_2_ [*I*]^0.74^ [*M*]^1.93^	97.25 ± 2.23
MADAAC	Amyl	*R*_p3_ = *k*_3_ [*I*]^0.75^ [*M*]^1.93^	100.23 ± 3.43

**Table 7:** Comparison of polymerization rates of different monomers.

No.	Monomer	[*M*] (mol·L^−1^)	[*I*] (mol·L^−1^)	*T* (℃)	Polymerization Rate
*R*_p1_	DMDAAC	2.5	0.02	50	*R*_p1_ > *R*_p2_ > *R*_p3_
*R*_p2_	MPDAAC	2.5	0.02	50	*R*_p1_ > *R*_p2_ > *R*_p3_
*R*_p3_	MADAAC	2.5	0.02	50	*R*_p1_ > *R*_p2_ > *R*_p3_

**Table 8:** Calculation results of polymerization rate constant *k*.

No.	Monomer	[*M*] (mol·L^−1^)	[*I*] (mol·L^−1^)	*k*	Polymerization Rate
*k*_1_	DMDAAC	1.92	0.72	0.036	0.0124
*k*_2_	MPDAAC	1.93	0.74	0.034	0.0108
*k*_3_	MADAAC	1.89	0.75	0.026	0.0079

## Data Availability

Data are contained within the article.

## Supplementary Materials

The following are available online at https://www.mdpi.com/article/10.3390/molecules27154677/s1, Table S1: The xyz-coordinates of the optimized structure of DMDAAC, Table S2: The xyz-coordinates of the optimized structure of MPDAAC, Table S3: The xyz-coordinates of the optimized structure of MADAAC, Table S4: Structural parameters of Methyl alkyl diallyl ammonium chloride monomers at the B3LYP/6-31G^*^ level, Table S5: Charge distribution of Methyl alkyl diallyl ammonium chloride at B3LYP/6-31G* level, Figure S1: FT-IR of MPDAAC (a) and PMPDAAC (b), Figure S2: FT-IR of MADAAC (a) and PMADAAC (b), Figure S3: FT-IR of MHDAAC (a) and PMHDAAC (b), Figure S4: ^1^H NMR of MPDAAC (a) and PMPDAAC (b), Figure S5: ^13^C NMR of MPDAAC (a) and PMPDAAC (b), Figure S6: ^1^H NMR of MADAAC (a) and PMADAAC (b), Figure S7: ^13^C NMR of MADAAC (a) and PMADAAC (b), Figure S8: ^1^H NMR of MHDAAC (a) and PMHDAAC (b), Figure S9: ^13^C NMR of MHDAAC (a) and PMHDAAC (b), Figure S10–S12: The conversion rates of each monomer under different reaction time and the relationships between monomer conversion rate and time.

## Sample Availability

Samples of the compounds are available from the authors X.Z. and X.J.
