# Supplementary material for: Effect of Substituents on the Homopolymerization Activity of Methyl Alkyl Diallyl Ammonium Chloride
Source: Molecules. 2022 Jul 22;27(15):4677. doi: 10.3390/molecules27154677 (PMC9330561; doi:10.3390/molecules27154677)
Supplement: Supplementary file 1 [file molecules-27-04677-s001.zip › molecules-1755993-supplementary.pdf]

Table S1. The xyz-coordinates of the optimized structure of DMDAAC.

Center Number	Atomic Number	Atomic Type	Coordinates (Angstroms)		
			X	Y	Z
1	6	0	−1.097062	0.781065	1.858283
2	6	0	−0.231394	2.668779	−0.639904
3	6	0	−0.30507	1.269875	−1.182186
4	7	0	−1.446156	0.384818	−0.671947
5	6	0	−1.187024	−0.202223	0.73271
6	6	0	0.892039	3.176724	−0.131084
7	6	0	−1.539415	−0.812276	−1.593035
8	6	0	−2.764678	1.10383	−0.703973
9	6	0	−0.031652	0.865687	2.657205
10	1	0	−1.993381	1.355775	2.0883
11	1	0	−1.104298	3.306092	−0.777397
12	1	0	0.625882	0.728117	−1.005836
13	1	0	−0.473776	1.315237	−2.26563
14	1	0	−2.042809	−0.877745	0.883898
15	1	0	−0.263311	−0.775848	0.636338
16	1	0	0.944871	4.220989	0.169773
17	1	0	1.780262	2.562322	−0.004103
18	1	0	−2.357476	−1.444891	−1.229193
19	1	0	−0.574675	−1.321731	−1.573136
20	1	0	−1.75889	−0.448264	−2.599894
21	1	0	−3.544668	0.378269	−0.426336
22	1	0	−2.919354	1.481943	−1.718565
23	1	0	−2.735566	1.936447	−0.002166
24	1	0	−0.033919	1.519153	3.526708
25	1	0	0.876266	0.29544	2.472042
26	17	0	−4.398687	−1.704551	0.253596

Table S2. The xyz-coordinates of the optimized structure of MPDAAC.

	Atomic Number	Atomic Type	Coordinates (Angstroms)		
			X	Y	Z
1	6	0	−1.290512	−2.024012	−1.261147
2	6	0	0.15612	−2.039911	1.794575
3	6	0	−0.126155	−0.603192	1.456746
4	7	0	−1.413197	−0.313398	0.675318
5	6	0	−1.260033	−0.584823	−0.839719
6	6	0	1.284187	−2.653552	1.434229
7	6	0	−1.769336	1.169575	0.843687
8	6	0	−2.575965	−1.090794	1.22458
9	6	0	−0.243445	−2.631759	−1.822107
10	1	0	−2.255553	−2.525522	−1.20865
11	1	0	−0.552798	−2.549685	2.446231
12	1	0	0.696293	−0.180891	0.881333
13	1	0	−0.237332	−0.030808	2.385687
14	1	0	−2.121413	−0.066662	−1.281729
15	1	0	−0.314869	−0.124701	−1.126722
16	1	0	1.501656	−3.664855	1.77066
17	1	0	2.015412	−2.17175	0.790682
18	1	0	−2.750013	1.279364	0.360989
19	1	0	−1.885222	1.313039	1.924424
20	1	0	−3.470596	−0.792117	0.659548
21	1	0	−2.67991	−0.843513	2.284859
22	1	0	−2.376529	−2.15531	1.111851
23	1	0	−0.324025	−3.644412	−2.211666
24	1	0	0.722824	−2.138849	−1.898925
25	17	0	−4.649179	0.47407	−1.012758
26	6	0	−0.763089	2.171307	0.279208
27	1	0	−0.712672	2.074189	−0.809827
28	1	0	0.250499	2.00675	0.659023
29	6	0	−1.221339	3.595284	0.625749
30	1	0	−0.529037	4.33021	0.20199
31	1	0	−2.222114	3.798552	0.227575
32	1	0	−1.250796	3.754292	1.71145

Table S3. The xyz-coordinates of the optimized structure of MADAAC.

Center Number	AtomicNumber	Atomic Type	Coordinates (Angstroms)		
			X	Y	Z
1	6	0	0.347705	−2.382572	−1.416548
2	6	0	1.436829	−2.242137	1.805006
3	6	0	0.55472	−1.046778	1.57793
4	7	0	−0.667616	−1.243892	0.673839
5	6	0	−0.321515	−1.172663	−0.833488
6	6	0	2.736264	−2.249848	1.504334
7	6	0	−1.687291	−0.134991	0.966461
8	6	0	−1.363784	−2.5422	0.97311
9	6	0	1.598774	−2.36667	−1.8808
10	1	0	−0.279867	−3.260765	−1.560289
11	1	0	0.996607	−3.088441	2.331643
12	1	0	1.129056	−0.209722	1.178384
13	1	0	0.127884	−0.733214	2.538963
14	1	0	−1.299548	−1.037537	−1.313084
15	1	0	0.302877	−0.287677	−0.961201
16	1	0	3.358227	−3.104327	1.763751
17	1	0	3.212265	−1.424927	0.976944
18	1	0	−2.550808	−0.386798	0.337991
19	1	0	−1.9695	−0.277896	2.016299
20	1	0	−2.266848	−2.585647	0.348272
21	1	0	−1.624604	−2.55225	2.034969
22	1	0	−0.687188	−3.366023	0.751074
23	1	0	2.017756	−3.236276	−2.383158
24	1	0	2.248795	−1.501813	−1.763351
25	17	0	−3.884712	−1.676858	−1.219608
26	6	0	−1.209292	1.299677	0.744778
27	1	0	−0.811464	1.434093	−0.265106
28	1	0	−0.390175	1.551631	1.426548
29	6	0	−2.37734	2.284835	0.954932
30	1	0	−1.955183	3.292049	1.075447
31	1	0	−2.888464	2.057115	1.90417
32	6	0	−3.404222	2.306516	−0.188698
33	1	0	−3.804056	1.301507	−0.373662
34	1	0	−2.889482	2.599214	−1.114548
35	6	0	−4.559718	3.276585	0.077895
36	1	0	−5.266067	3.290127	−0.760178
37	1	0	−5.120369	2.988603	0.976886
38	1	0	−4.197904	4.302713	0.228936

Table S4. Structural parameters of Methyl alkyl diallyl ammonium chloride monomers at the B3LYP/6-31G* level.

DMDAAC				MPDAAC				MADAAC			
C/No.	Å	C/No.	°	C/No.	Å	C/No.	°	C/No.	Å	C/No.	°
C9-C1	1.33	N4-C5-C1	116.08	C29-C26	1.53	C7-C26-C29	109.10	C35-C32	1.53	C29-C32-C35	112.35
C8-N4	1.50	C3-N4-C5	112.41	C26-C7	1.52	N4-C7-C26	116.55	C32-C29	1.53	C26-C29-C32	114.22
C7-N4	1.51	C3-N4-C7	108.36	C9-C1	1.33	N4-C5-C1	115.88	C29-C26	1.54	C7-C26-H27	111.38
C6-C2	1.33	C3-N4-8	112.18	C8-N4	1.50	C3-N4-C5	112.14	C26-C7	1.52	C7-C26-H28	110.93
C5-C1	1.49	C5-N4-C7	105.99	C7-N4	1.53	C3-N4-C7	109.63	C9-C1	1.33	C7-C26-C29	109.96
C5-N4	1.54	C5-N4-C8	109.94	C6-C2	1.33	C3-N4-8	111.89	C8-N4	1.50	N4-C7-C26	116.74
N4-C3	1.53	C7-N4-C8	107.61	C5-C1	1.49	C5-N4-C7	108.04	C7-N4	1.54	N4-C5-C1	116.18
C3-C2	1.50	C2-C3-N4	116.84	C5-N4	1.54	C5-N4-C8	109.09	C6-C2	1.33	C3-N4-C5	112.62
N4-Cl26	3.59	C3-C2-C6	123.77	N4-C3	1.53	C7-N4-C8	105.76	C5-C1	1.49	C3-N4-C7	109.58
		C5-C1-C9	122.78	C3-C2	1.50	C2-C3-N4	116.69	C5-N4	1.54	C3-N4-8	111.76
				N4-Cl25	3.62	C3-C2-C6	123.80	N4-C3	1.52	C5-N4-C7	107.74
						C5-C1-C9	122.70	C3-C2	1.50	C5-N4-C8	108.97
								N4-Cl25	3.63	C7-N4-C8	105.85

Table S5. Charge distribution of Methyl alkyl diallyl ammonium chloride at B3LYP/6-31G* level.

DMDAAC			MPDAAC			MADAAC		
No.	Atom	Charge density	No.	Atom	Charge density	No.	Atom	Charge density
1	C	-0.051	1	C	-0.051	1	C	−0.051
2	C	-0.067	2	C	-0.068	2	C	−0.068
3	C	-0.176	3	C	-0.178	3	C	−0.177
4	N	-0.362	4	N	-0.378	4	N	−0.378
5	C	-0.217	5	C	-0.223	5	C	−0.222
6	C	-0.313	6	C	-0.315	6	C	−0.314
7	C	-0.339	7	C	-0.157	7	C	−0.164
8	C	-0.349	8	C	-0.348	8	C	−0.348
9	C	-0.338	9	C	-0.339	9	C	−0.338
10	H	0.157	10	H	0.158	10	H	0.157
11	H	0.156	11	H	0.154	11	H	0.154
12	H	0.183	12	H	0.185	12	H	0.185
13	H	0.184	13	H	0.183	13	H	0.184
14	H	0.264	14	H	0.262	14	H	0.262
15	H	0.159	15	H	0.163	15	H	0.163
16	H	0.166	16	H	0.164	16	H	0.164
17	H	0.161	17	H	0.160	17	H	0.159
18	H	0.268	18	H	0.253	18	H	0.252
19	H	0.169	19	H	0.152	19	H	0.154
20	H	0.171	20	H	0.266	20	H	0.266
21	H	0.268	21	H	0.166	21	H	0.167
22	H	0.166	22	H	0.181	22	H	0.181
23	H	0.182	23	H	0.157	23	H	0.157
24	H	0.158	24	H	0.145	24	H	0.145
25	H	0.146	25	Cl	-0.750	25	Cl	−0.746
26	Cl	-0.747	26	C	-0.291	26	C	−0.294
			27	H	0.173	27	H	0.157
			28	H	0.136	28	H	0.132
			29	C	-0.455	29	C	−0.258
			30	H	0.155	30	H	0.134
			31	H	0.190	31	H	0.133
			32	H	0.145	32	C	−0.271
						33	H	0.205

34	H	0.128
35	C	−0.443
36	H	0.155
37	H	0.142
38	H	0.130

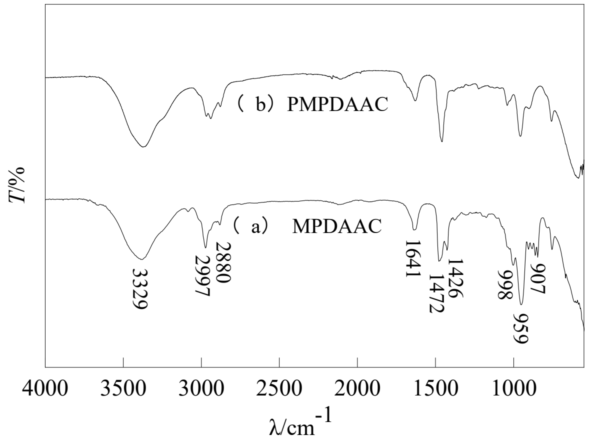

Figure S1. FT-IR of MPDAAC (**a**) and PMPDAAC (**b**).

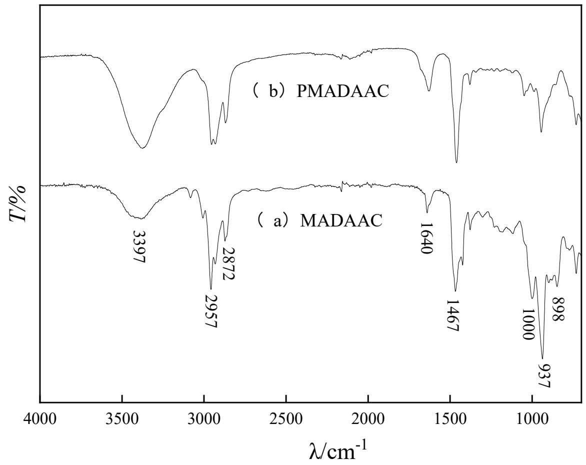

Figure S2. FT-IR of MADAAC (**a**) and PMADAAC (**b**).

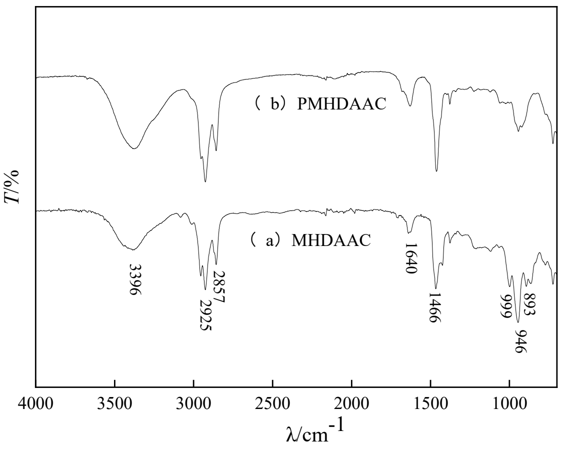

Figure S3. FT-IR of MHDAAC (**a**) and PMHDAAC (**b**).

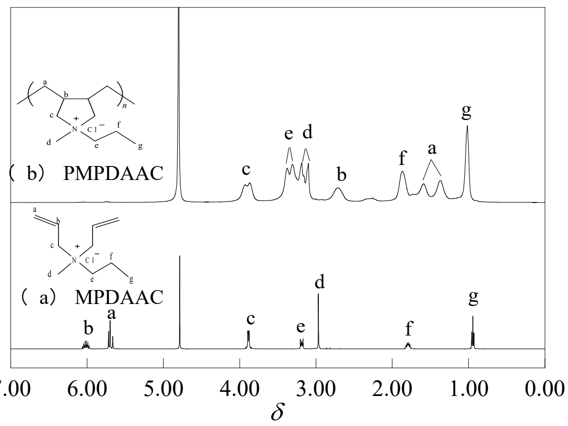

Figure S4. ^{1}H NMR of MPDAAC (**a**) and PMPDAAC (**b**).

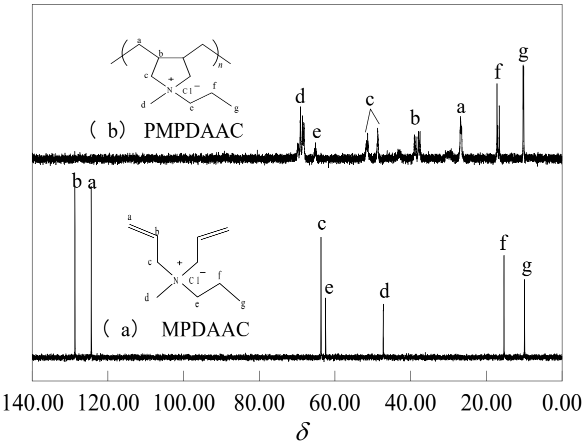

Figure S5. ^{13}C NMR of MPDAAC (**a**) and PMPDAAC (**b**).

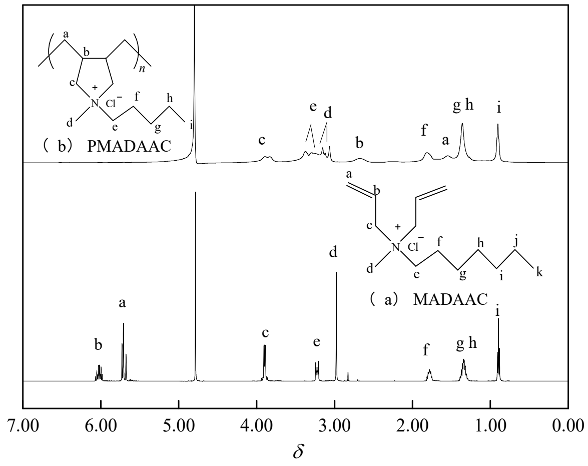

Figure S6. ^{1}H NMR of MADAAC (**a**) and PMADAAC (**b**).

.

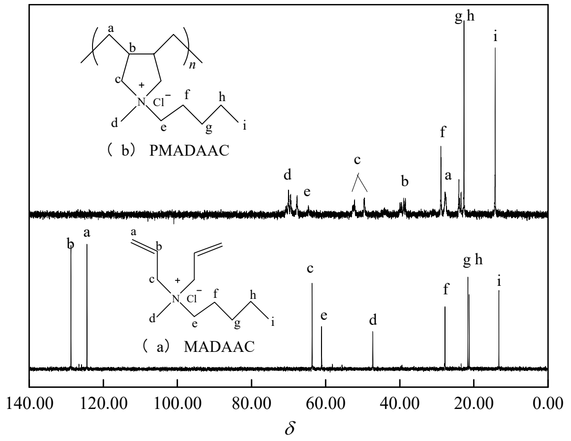

Figure S7. ^{13}C NMR of MADAAC (**a**) and PMADAAC (**b**).

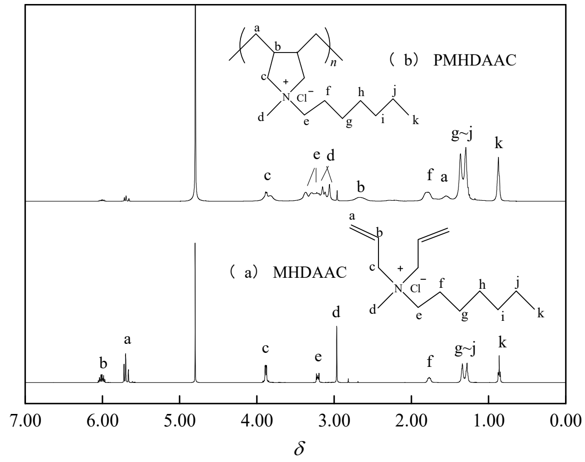

Figure S8. ^{1}H NMR of MHDAAC (**a**) and PMHDAAC (**b**).

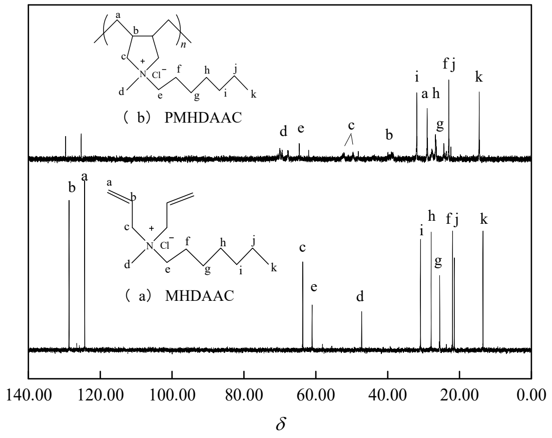

Figure S9. ^{13}C NMR of MHDAAC (**a**) and PMHDAAC (**b**).

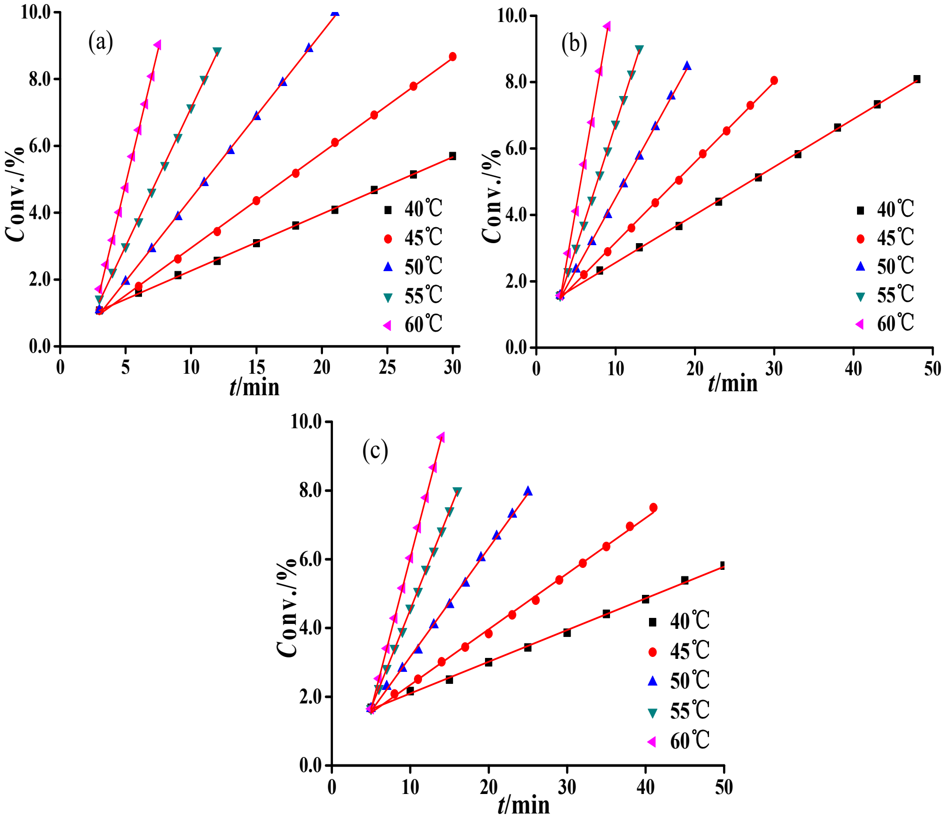

Figure S10. The fixed process conditions: [M]=2.5 mol/L, [I]=0.02 mol/L, when the polymerization temperature were 40°C, 45°C, 50°C, 55°C and 60°C, respectively, the conversion rate of each monomer under different reaction time and the relationships between monomer conversion rate and time were obtained (Conv.%-t diagram). (**a**) DMDAAC, (**b**) MPDAAC, (**c**) MADAAC.

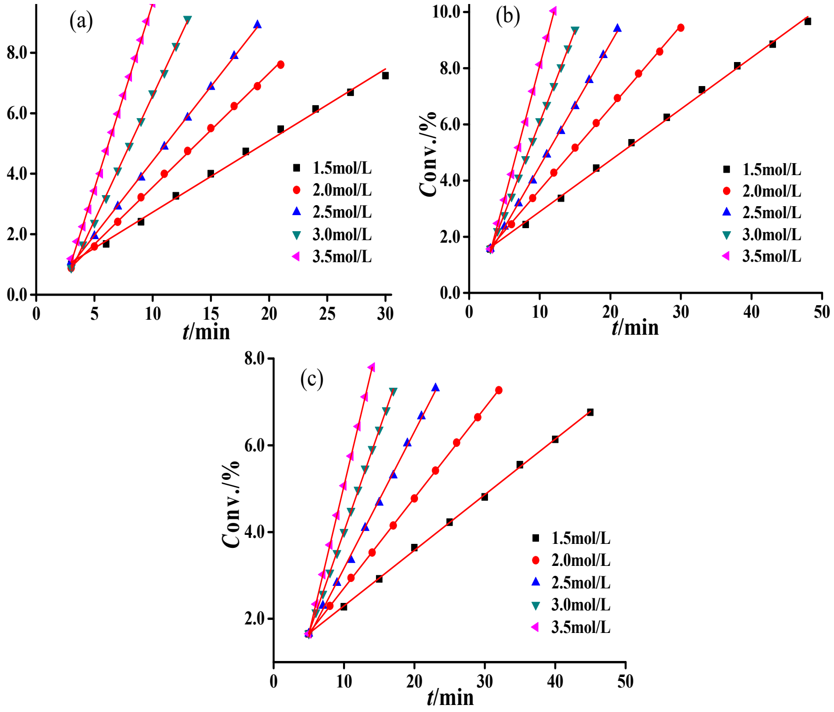

Figure S11. The fixed process conditions: [*I*]=0.02 mol/L, reaction temperature was 50 °C, when the concentration of each monomer was 1.5 mol/L, 2 mol/L, 2.5 mol/L, 3 mol/L and 3.5 mol/L respectively, the conversion rate of each monomer under different reaction time and the relationships between monomer conversion rate and time were obtained (*Conv.*%-t diagram). (**a**) DMDAAC, (**b**) MPDAAC, (**c**) MADAAC.

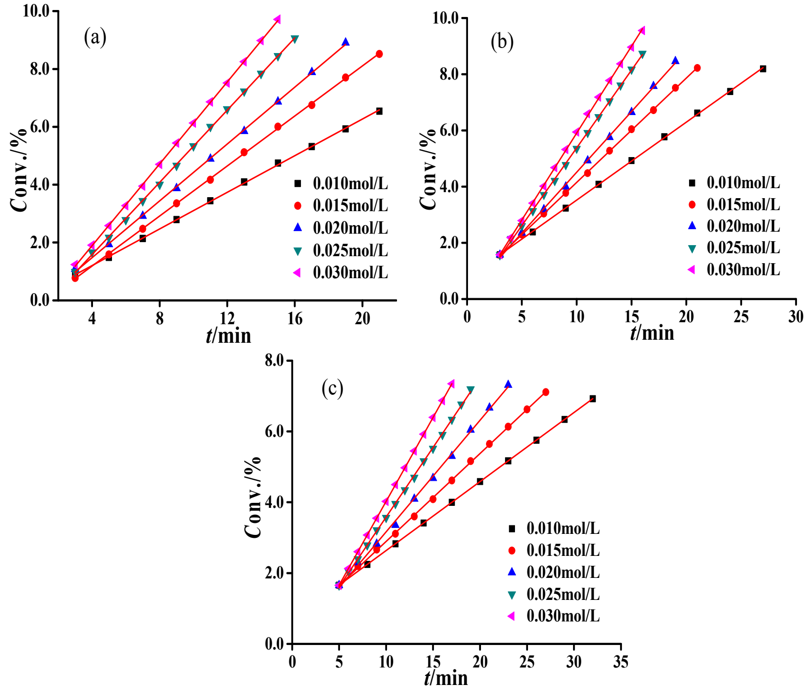

Figure S12. The fixed process conditions: [*M*]=2.5 mol/L, reaction temperature was 50℃, when the concentration indices of initiator were 0.01 mol/L, 0.015 mol/L, 0.02 mol/L, 0.025 mol/L and 0.03 mol/L, the conversion rate of each monomer under different reaction time and the relationships between monomer conversion rate and time were obtained (*Conv.*%-t diagram). (**a**) DMDAAC, (**b**) MPDAAC, (**c**) MADAAC.
